# ESCRT may function as a tumor biomarker, transitioning from pan-cancer analysis to validation within breast cancer

**DOI:** 10.3389/fimmu.2025.1531940

**Published:** 2025-03-31

**Authors:** Xiao-rui Chen, Xue-ying Tan, Zong-liang Zhang, Jiang-shui Yuan, Wei-qing Song

**Affiliations:** ^1^ Qingdao Medical College, Qingdao University, Qingdao, China; ^2^ Clinical Laboratory Department, Qingdao Municipal Hospital, Qingdao, China; ^3^ Department of Urology, Affiliated Hospital of Qingdao University, Qingdao, China

**Keywords:** pan-cancer, ESCRT, biomarker, immune cell infiltration, VPS37D

## Abstract

**Background:**

Studies have shown that ESCRT genes affect cell aging, immune environment, and tumors. BRCA was used to explore its effects on tumor prognosis and therapy.

**Methods:**

The data sets of Cancer Genome Atlas (TCGA), Genome-Tissue Expression Plan (GTEX), Human Protein Mapping (HPA), Gene Expression Omnibus (GEO), Clinical Proteomic Tumor Analysis Consortium (CPTAC), R software package, and bioinformatics methods were used to mine the potential carcinogenic effects of ESCRT, including the expression level, prognostic value, and immune value of ESCRT in various types of tumor tissues, and the potential function of ESCRT family genes was further verified in breast cancer.

**Results:**

ESCRT showed significant differential expression in various cancers, such as bladder urothelial carcinoma and liver, cervical, renal, esophageal, head, and neck cancers (*P <*0.05). Abnormal ESCRT expression is associated with poor prognosis in various cancers, such as adrenocortical carcinoma, bladder urothelial carcinoma, breast cancer, and cervical cancer (*P <*0.05). The expression level of ESCRT was significantly associated with immune cell infiltration and the modulation of the stromal/immune score (all *P <*0.05). Enrichment analysis showed that ESCRT is associated with immune-related functions and transport signaling pathways in various tumor cells. Moreover, ESCRT serves as an early diagnostic marker for several tumors and is significantly associated with prognosis. This confirms that ESCRT is associated with most immune-infiltrating cells in pan-carcinomas. Taken together, these studies highlight the importance of the ESCRT family gene VPS37D in breast cancer initiation, progression, and immune response.

**Conclusion:**

This study highlights ESCRT’s potential in tumor detection via pan-cancer analysis, showing expression variations between tumor and normal tissues, its role in cancer progression through the immune microenvironment, and its specificity and sensitivity in detection. The VPS37D gene, with significant variation in breast cancer, predicts patient prognosis and is related to the tumor microenvironment, suggesting that ESCRT is a novel biomarker for early diagnosis and prognosis assessment.

## Introduction

Despite advancements in surgery and treatment, tumor incidence and mortality rates remain high (1). Tumor development is influenced by genetic factors, such as oncogene mutations (2), epigenetic changes affecting gene expression, and an imbalance between cell proliferation and apoptosis (3). Environmental and lifestyle factors such as smoking and exposure to industrial chemicals also contribute to this phenomenon (4). Chronic inflammation caused by conditions such as recurrent urinary tract infections can damage bladder cells and increase the risk of cancer (5). Tumor growth requires angiogenesis, and cancer cells can metastasize through lymphatic or blood pathways, thereby worsening prognosis. Additionally, tumor cells may evade immune surveillance, facilitating their growth and spread (6). Cancer treatments include surgery for non-muscle-invasive cancers (7), chemotherapy at various stages (8, 9), radiotherapy as an adjunct for advanced or inoperable cases (10), immunotherapy to activate the immune system (11, 12), targeted therapy focusing on specific cancer cell pathways (13), palliative care for advanced cancers (14, 15), Traditional Chinese Medicine as supportive care (16), and combination therapy for optimal results, with ongoing research on new options (17, 18).

Breast cancer is the most prevalent type of cancer among women worldwide (19), with its increasing incidence presenting significant challenges to public health systems and healthcare resources (20). Consequently, early screening and timely treatment are critical for reducing mortality rates and enhancing cure rates (21), positioning breast cancer treatment as the central focus of contemporary medical research (22). The integration of various treatment modalities and emphasis on early intervention are essential strategies in the fight against this widespread disease (23).

**Figure 1:**
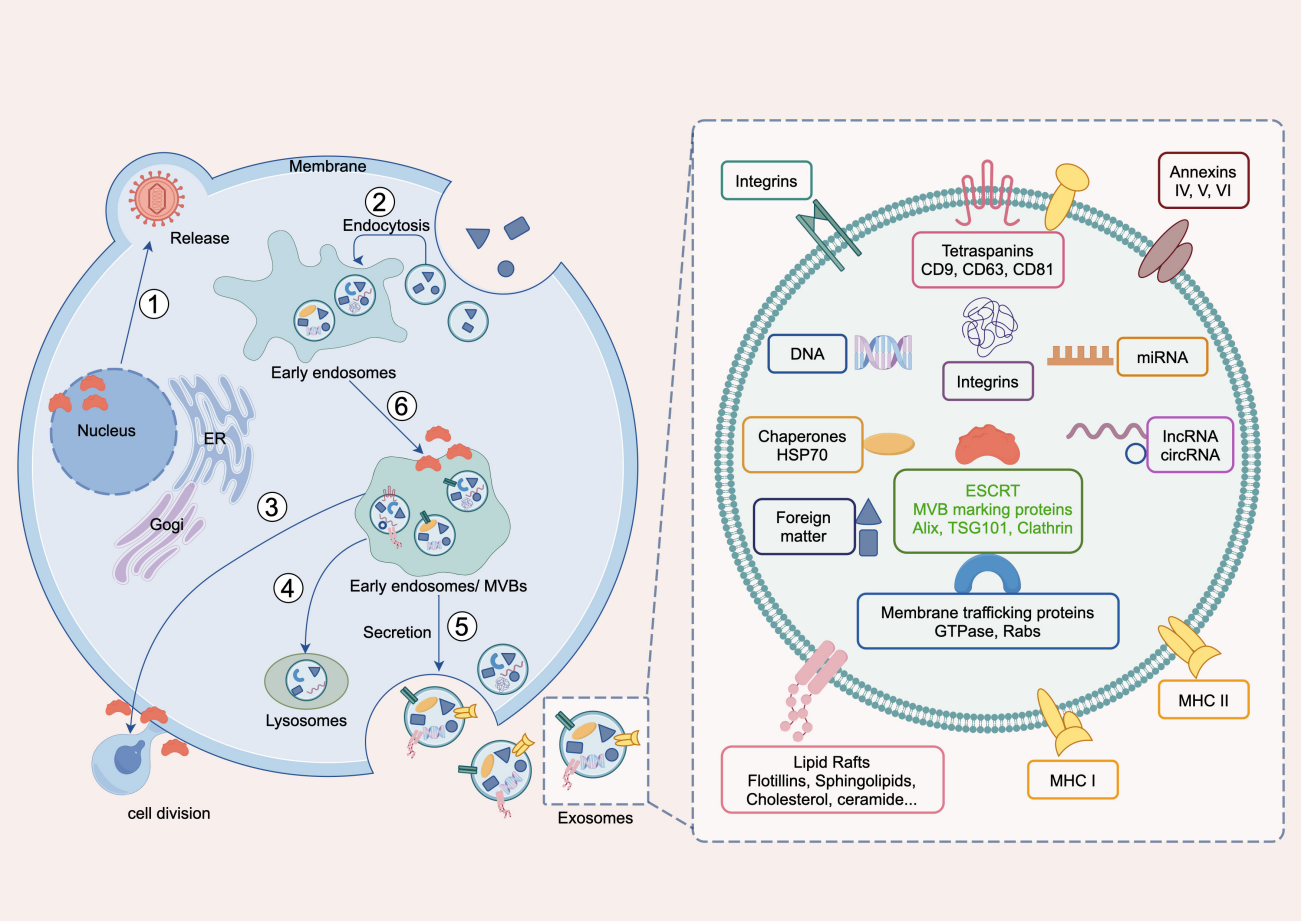
Main biological functions of ESCRT. ① Viral release in the nucleus, Maintaining nuclear pore stability, nuclear envelope repair and remodeling, virus secretion; ② endocytosis, endomorphism; ③ cell division; ④ Lysosomal membrane repair and autophagy; ⑤ exdosomes secretion, ILV formation; and ⑥ MVB formation, microautophagy.

In recent years, the endosomal sorting transport complex (ESCRT) has emerged as a fundamental molecular apparatus for regulating membrane dynamics (24) and cellular signal transduction (25), and its role in cancer mechanisms has gained increasing attention (26, 27). It is composed of ESCRT-0, ESCRT-I, ESCRT-II, ESCRT-III, VPS4-VTA1 and ALIX (28) (**Supplementary Figure S1**). They play key roles in processes, such as intracellular material transport (29), membrane repair (30, 31), and cell division (32) (**Figure 1**). ESCRT is linked to cancer development through several mechanisms, including regulating cell cycle proteins affecting tumor cell survival (33), facilitating protein transport for degradation (34), influencing apoptosis and autophagy (35), modulating tumor-immune interactions (36), assisting exosome formation impacting the tumor microenvironment (37), participating in DNA damage repair (38), and affecting cancer stem cell behavior (39), highlighting its crucial role in tumorigenesis and progression (40). Previous studies have highlighted ESCRT’s role in tumors and its therapeutic strategies (41), indicating its significance in hepatocellular carcinoma (42), breast cancer (43), and renal cancer (44). A previous study also predicted the impact of ESCRT family gene expression on the prognosis of BLCA. However, no pan-cancer analysis of ESCRT’s effect on cancer prognosis has been conducted (45). Such analyses enhance the understanding of gene mechanisms and their predictive value in tumor biology, aiding clinical diagnosis and treatment (46).

This study represents an inaugural multidimensional analysis of ESCRT, elucidating its expression characteristics, prognostic associations, and immune regulatory mechanisms across 33 cancer types. The integration of tumor mutation load, microsatellite instability, and stem cell score revealed a significant correlation between ESCRT and the expression of immune checkpoint genes as well as immune cell infiltration. This study used breast cancer as a model to investigate the potential significance of VPS37D and other critical molecules in the remodeling of the tumor microenvironment and precision therapy, thereby offering a theoretical foundation for the advancement of tumor immunotherapy.

## Materials and methods

### Collection and processing of the ESCRT data

RNA sequencing data for the pan-cancer cohort (n = 15,776) were obtained from the UCSC Xena website (https://xenabrowser.net/datapages/). The dataset comprised of several cancerous and normal tissues from the Cancer Genome Atlas and genotype-tissue expression (GTEx). The complete dataset was filtered to eliminate missing and duplicate entries, and the logarithm (TPM + 1) of uniformly processed transcripts per million (TPM) normalized expression profile data was transformed using the rms function in the R package for subsequent analysis. CPTA data were obtained from PDC (https://pdc.cancer.gov/pdc/browse). Each expression value was log2 (value + 1) transformed, and cancers with fewer than five samples in a single cancer species were excluded. Finally, the expression data of 33 tumors were obtained, including adrenocortical carcinoma (ACC), bladder urothelial carcinoma (BLCA), breast invasive carcinoma (BRCA), cervical squamous cell carcinoma and endocervical adenocarcinoma (CESC), cholangiocarcinoma (CHOL), colon adenocarcinoma (COAD), lymphoid neoplasm diffuse large B-cell lymphoma (DLBC), esophageal carcinoma (ESCA), glioblastoma multiforme (GBM), head and neck cancer (HNSC), kidney chromophobe (KICH), kidney renal clear cell carcinoma (KIRC), kidney renal papillary cell carcinoma (KIRP), acute myeloid leukemia (LAML), brain lower grade glioma (LGG), liver hepatocellular carcinoma (LIHC), lung adenocarcinoma (LUAD), lung squamous cell carcinoma (LUSC), mesothelioma (MESO), ovarian serous cystadenocarcinoma (OV), pancreatic adenocarcinoma (PAAD), pheochromocytoma and paraganglioma (PCPG), prostate adenocarcinoma (PRAD), rectum adenocarcinoma (READ), sarcoma (SARC), skin cutaneous melanoma (SKCM), stomach adenocarcinoma (STAD), testicular germ cell tumors (TGCT), thyroid carcinoma (THCA), thymoma (THYM), uterine corpus endometrial carcinoma (UCEC), uterine carcinosarcoma (UCS) and uveal melanoma (UVM).

### ESCRT differential expression analysis

After excluding cancers with fewer than five samples from a single cancer species, we included the data for 18 tumors. The DESeq2 package in R (4.2.3) analyzes transcript data from normal and cancer tissues, filtering differentially expressed genes (DEGs) with log2|FC| >0.585 and false discovery rates (FDR)-adjusted *P*-value <0.05, intersects them with ESCRT, and visualizes results using “ggot2” and “VennDiagram” for a Venn diagram of ESCRT genes. It also employs “pubggr” and “pheatmap” for volcano and heatmap visualizations, while “ggpubr” creates a box plot of differential gene expression and outputs significance from Wilcoxon testing. We also used UALCAN (https://ualcan.path.uab.edu/index.html) to process CPTAC data and analyze the differential protein expression of the ESCRT family genes in pan-cancer. Bonferroni Correction for p <0.05 was considered as having a significant difference.

### Analysis of prospective relevance

Forecasting of patients using the ESCRT family gene expression median in TCGA cohort as critical values, using log-rank testing to analyze the correlation of ESCRT family genetic expression with total survival (OS) in pan-cancer patients, and to map the Kaplan–Meier survival curve.

### Correlation analysis of immune traits

An immunogenicity analysis of 33 cancers in TCGA identified six immune subtypes: wound healing, IFN-γ dominant, inflammatory, lymphocyte-depleted, immunologically quiet, and TGF-β dominant, comparing ESCRT genes among them. All results were subjected to multiple validations, and the P-adjust was calculated using the Bonferroni correction. The relationship between ESCRT family gene expression and immune cell infiltration was analyzed using multiple algorithms. In the Spearman correlation, statistical significance was determined by a *P*-value <0.05 and the correlation between ESCRT and immune cell infiltration was visualized using the R packages ‘ggplot2’ and ‘ggpubr.’

### Association of ESCRT with stemness

The OCLR algorithm produces the DNAss index by analyzing the methylation patterns of different types of cancer. This was used to determine the stemness score of tumors. The stemness scores and gene expression data were integrated to visualize the Pearson correlation. Raw *P* values were adjusted using Bonferroni’s method.

### Drug sensitivity analysis of ESCRT

The ESCRT transcriptomic and drug sensitivity data were downloaded from CellMiner40 and a Pearson correlation test was used to analyze their relationships. The impute and limma packages identified the link between ESCRT expression and drug sensitivity, whereas ggplot2 and ggpubr were used for visualization.

### Differential expression analysis in BRCA

Using transcriptomic data from TCGA, GTEx, GEO (GSE42568, GSE20685), Clinical Proteomic Tumor Analysis Consortium (CPTAC), and Human Protein Atlas (HPA) databases, multi-omics approaches were employed to compare the expression levels of ESCRT genes in BRCA and normal tissues, identifying the most significant key genes. Set |log2FC| >0.585 and adjusted *P*-value <0.05. For multi-center or cross-platform data, the ComBat algorithm (sva package) was used to eliminate technical batch differences.

### GO function enrichment set of BRCA differential genes and KEGG signal path analysis

The “clusterprofiler” package “org.HS.eg.7db” was used to screen for differentially expressed genes with a significance threshold of P <0.05. This includes conducting Gene Ontology (GO) and Kyoto Encyclopedia of Genes and Genomes (KEGG) enrichment analyses. GO analysis was categorized into three distinct components: biological process (BP), molecular function (MF), and cellular component (CC). Following the examination, the downloaded results were processed by applying filtering conditions with *p.adjust <*0.05, and FDR <0.25. The combined results were subsequently represented through the ‘ggplot2’ software.

### Relevance analysis of clinical characteristics

#### Exploring the expression of the key gene in BRCA: VPS37D in different TNM classifications and pathological categories

##### Tumor microenvironment in BRCA

Obtaining breast cancer (BRCA) RNA-seq data from the public database TCGA was normalized to FPKM (RNA-seq). The ESTIMATE algorithm was used to calculate the stromal and immune scores and the integrated ESTIMATE score (reflecting tumor purity) of tumor samples. For RNA-seq data based on the LM 22 feature matrix, the relative proportions of the 22 immune cell subtypes were calculated, keeping only samples with a deconvolved *P*.adjust <0.05, FDR <0.25. We estimated the abundance of 64 immune and stromal cell types using gene set enrichment analysis (GSEA) and the extended ssGSEA method to produce xCell.

## Results

### Expression analysis of the ESCRT family genes

**Figure 2:**
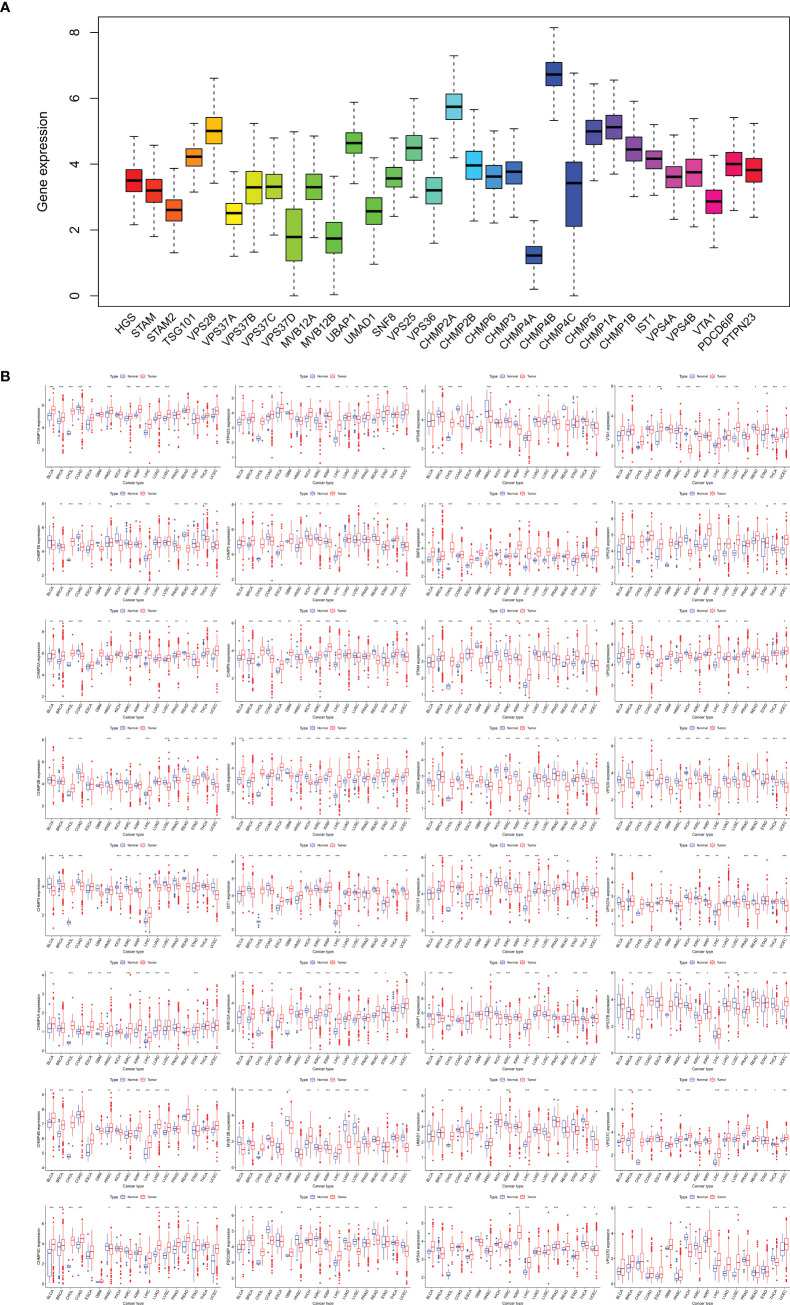
ESCRT family gene expression. **(A)** Gene expression levels in pan-cancer patients; **(B)** Differential expression of ESCRT between 18 tumor and normal tissue samples (*P <0.05, **P <0.01, ***P <0.001).

The expression levels of the ESCRT family genes in 18 tumors and their corresponding normal tissues were analyzed using TCGA (**Figure 2A**). Overall, ESCRT genes were significantly overexpressed in tumors compared to in normal tissues (all *P <*0.05). Individual analysis of each gene revealed some variations: compared to the corresponding normal tissues, all ESCRT family genes were highly expressed in CHOL, CHMP4A was significantly overexpressed in all cancers, HGS was low in GBM and KIRC, but high in other tumors; TSG101 was low in KIRC; VPS28 was low in KICH; VPS37A was high in CHOL and LIHC; VPS37C was low in THCA; VPS37D was high in BRCA; MVB12A was low in STAD and KIRC; SNF8 was low in KICH; VPS36 was high in CHOL; CHMP3 was high in KICH; CHMP4B was low in KICH, HNSC, and KIRC; CHMP1A was low in COAD; VPS4A was low in THCA and UCEC; and PTPN23 was low in KIRC and LUSC. All results were statistically significant (*P <*0.05) (**Figure 2B**). The differential expression of the ESCRT family genes is shown in **Supplementary Figure S2**.

### Analysis of the relationships between ESCRT and prognosis

**Figure 3:**
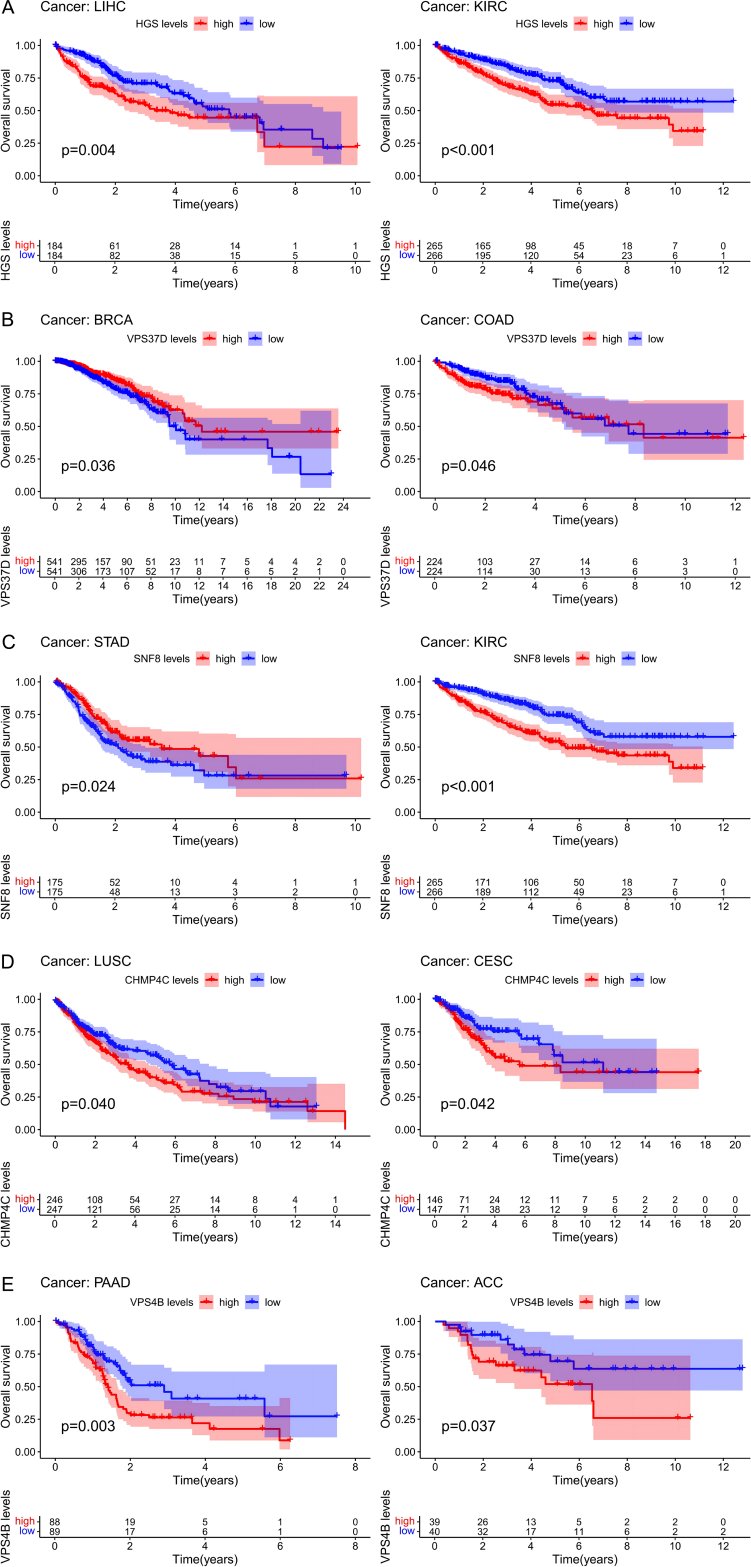
The prognosis value of ESCRT in pan-cancer. **(A)** Prognostic analysis of ESCRT-0 representative gene HGS in LIHC and KIRC; **(B)** Prognostic analysis of ESCRT-I representative gene VPS37D in BRCA and COAD; **(C)** ESCRT-II representative gene SNF 8 in STAD and KIRC; **(D)** ESCRT-III representative prognostic analysis of CHMP4C in LUSC and CESC; **(E)** Prognostic analysis of VPS 4-VTA 1 representative gene VPS4B in PAAD and ACC.

By analyzing the expression of the ESCRT family genes and their correlation with the overall survival of pan-cancer patients, we concluded that in all 33 cancer types, the ESCRT family genes were significantly associated with the prognosis of a certain tumor (**Supplementary Figure S3**; *P <*0.05). We selected the prognosis for one gene in common tumors in each ESCRT subcomplex classification. Genes such as HGS, CHMP4C, and VPS4B, which are highly expressed, are associated with poor prognosis. However, it is important to note that some genes, such as VPS37D and STAD, have different prognostic effects on different types of cancer, which may be because the tumors are not the same (*P <*0.05) (**Figure 3**).

### Immune correlation analysis

**Figure 4:**
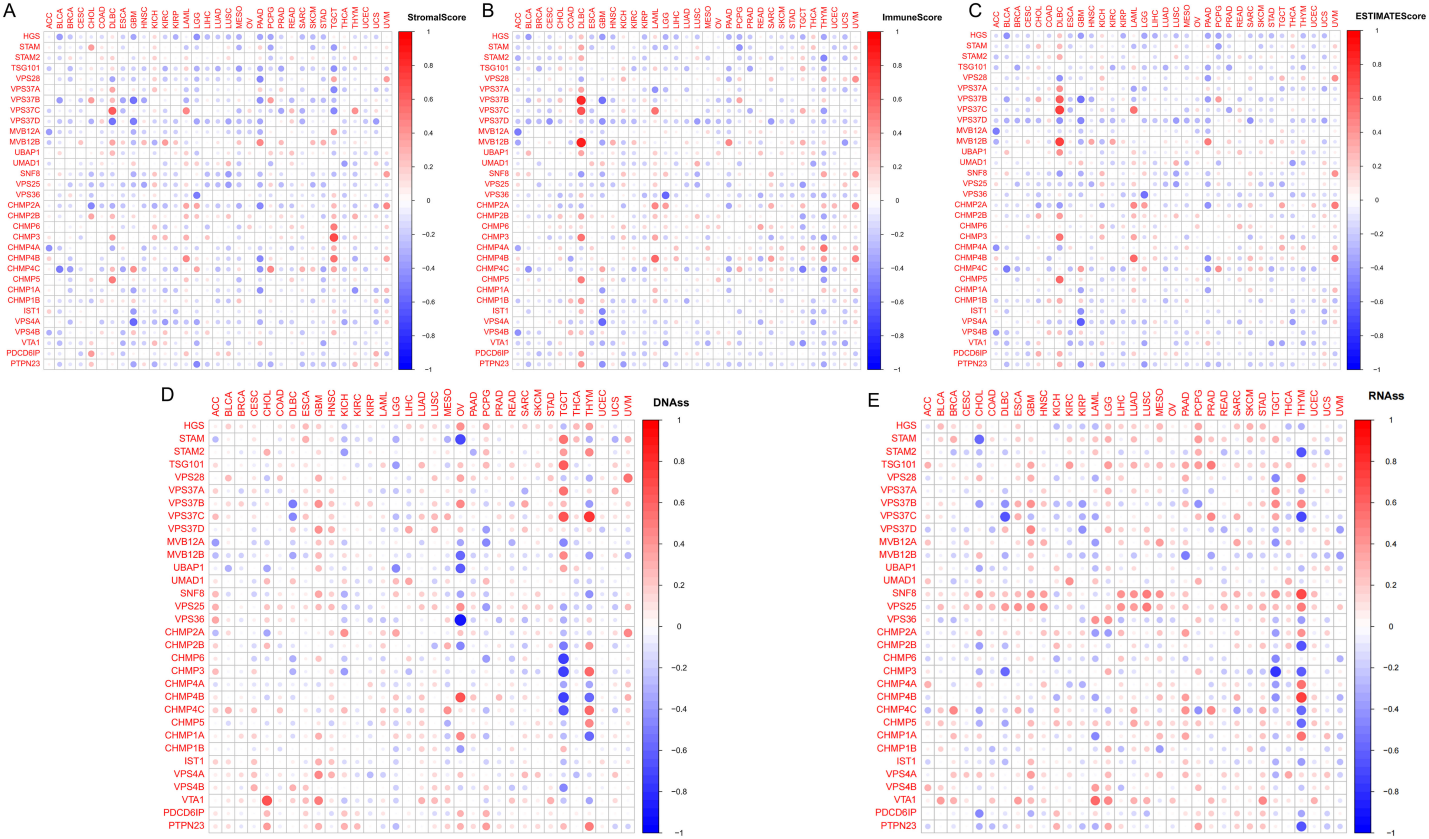
The correlation between ESCRT expression and immune infiltration. **(A)** Correlation of ESCRT expression with StromalScore; **(B)** Correlation of ESCRT expression with ImmuneScore; **(C)** Correlation of ESCRT expression with EstimateScore; and **(D, E)** Correlation of ESCRT expression with tumor cell stemness.

TCGA tumors were classified into six immune subtypes (C1: wound healing, C2: IFN-γ dominant, C3: inflammatory, C4: lymphocyte depleted, C5: immunologically quiet, and C6: TGF-β dominant), and the analysis showed that the ESCRT family genes were significantly different in all immune subtypes (**Supplementary Figure S4**). Evaluation using StromalScore, ImmuneScore, and EstimateScore indicated that most ESCRT genes, such as VPS37D in DLBC and VPS37B in GBM, exhibited a significant negative correlation with tumor immune infiltration (**Figures 4A–C**). Some genes exhibited positive regulation in particular cancer types, including VPS37C in DLBC and CHMP3 and VPS37B in TGCT, which were positively correlated with immune cell enrichment. This indicates that members of the ESCRT family may have immune regulatory functions specific to certain cancer species. Association of cancer stem cells: Integrated analysis of the stem cell transcriptome and DNA methylation multi-platform data indicates a strong correlation between ESCRT gene expression and cancer stem cell characteristics. In CHOL, genes VTA 1 and CHMP4B exhibited a positive correlation with the methylation-derived stem cell index, whereas CHMP6 in STAM and TGCT showed a significant negative correlation (**Figures 4D, E**). The RNA dataset confirmed that CHMP4B/SNF8 in THYM positively regulates stem cell properties, whereas VPS37C in DLBC and CHMP3 in TGCT negatively influence this process. This indicates that ESCRT impacts the biological behavior of tumor stem cells through a dual mechanism involving epigenetic and transcriptional regulation.

### Drug sensitivity analysis

**Figure 5:**
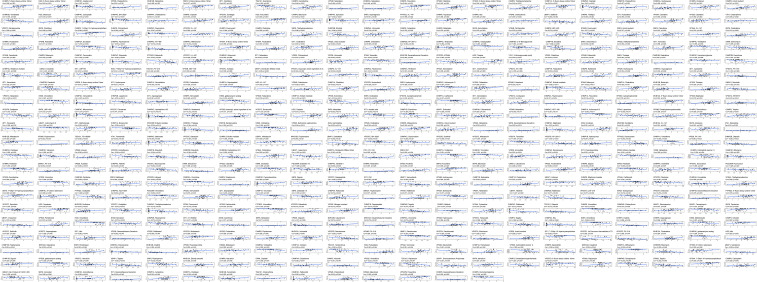
Correlation analysis of ESCRT expression and antitumor drug sensitivity.

Systemic drug therapies play a crucial role in cancer treatment. Gene expression and drug sensitivity data were obtained using CellMiner. We screened FDA-approved drugs and analyzed the correlation coefficients between ESCRT expression and drug sensitivity to investigate the relationship between ESCRT and anti-tumor drugs. The findings indicated 434 significant drug-sensitivity relationships, notably between CHMP6 and 5-fluoro deoxy uridine 10mer. An increase in CHMP6 expression was correlated with enhanced sensitivity to 5-fluoro deoxy uridine 10mer. The expression of HGS correlated positively with drug sensitivity, whereas CHMP4C exhibited an inverse correlation with sensitivity to okadaic acid. Additionally, VPS37D expression was positively correlated with Sonidegib, 5-fluoro deoxy uridine 10mer, and the 1st Precursor Intermediate to TDP 665759, but negatively correlated with Cyclophosphamide, Sunitinib, Obatoclax, and PX-316 (**Figure 5**). In conclusion, the ESCRT gene family demonstrated a significant correlation with sensitivity to various drugs. These results indicate that sensitization to specific drugs may be influenced by the expression of ESCRT family genes, either by facilitating or inhibiting the process.

### Differential expression of ESCRT family genes in BRCA

**Figure 6:**
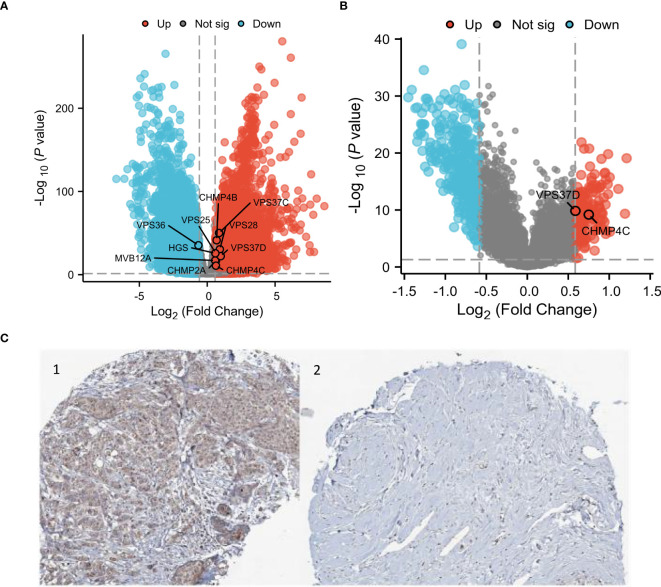
Differential analysis of the ESCRT family genes in breast cancer. **(A)** Heatmap of the ESCRT family genes; **(B)** Volcanic map of differential genes; and **(C)** VPS37D immunosynthesis. 1.BRCA Organization 2. Normal breast tissue.

The results showed differential expression of ESCRT family genes in breast cancer. We subsequently analyzed data from TCGA and GEO to examine the key molecules associated with BRCA. TCGA analysis revealed 8,576 differentially expressed genes in both BRCA and normal tissues, and when intersected with the ESCRT gene family, 11 DEGs were identified, including HGS, MVB12A, VPS28, VPS36, VPS37C, VPS37D, VPS 25, CHMP2A, CHMP4B, and CHMP4C, with VPS37D showing the most significant difference in breast cancer (P.adjust <0.05, **Figure 6A**). We subsequently validated the protein expression of VPS37D in the HPA database, and the results showed that it was highly expressed in breast cancer tissues compared to normal breast tissues (*P <*0.05, **Figure 6C**). We identified 505 differentially expressed genes using GEO analysis (*P <*0.05, **Figure 6B**).

### Enrichment analysis

**Figure 7:**
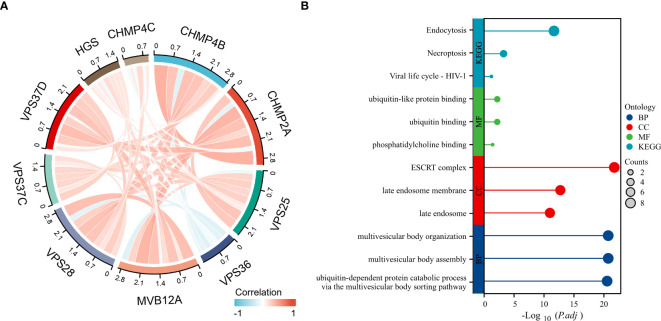
Enrichment analysis of significantly differential genes. **(A)** Differential genetic relationship chart; **(B)** Results of differential gene enrichment analysis.

Key genes identified by TCGA differential analysis were also closely related (**Figure 7A**). KEGG enrichment analysis of differentially expressed genes indicated significant enrichment in pathways, including endocytosis, viral life cycle-HIV-1 and necroptosis. GO enrichment analysis revealed that these genes were mainly involved in molecular functions, such as ubiquitin binding, ubiquitin-like protein binding, and phosphatidylinositol binding. Cellular components mainly involve the late endosome membrane and late endosomes. Moreover, these genes play crucial roles in biological processes, including multivesicular body assembly and organization (**Figure 7B**).

### Differential analysis of the clinical correlation

**Figure 8:**
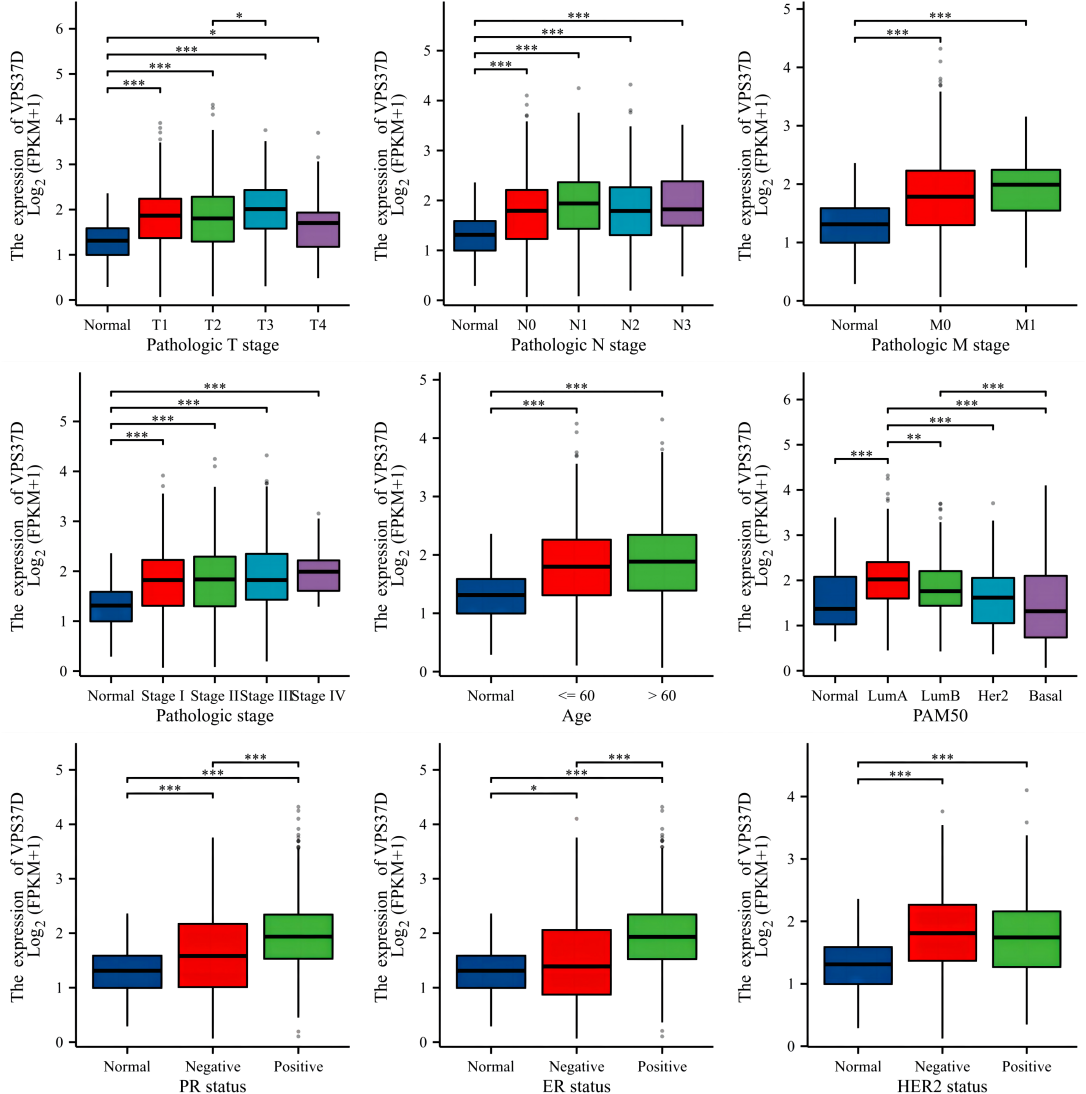
Correlation analysis between VPS37D expression and BRCA clinical characteristics (*P <0.05, **P <0.01, ***P <0.001).

By combining the differential gene VPS37D with clinical analysis, it was observed that there were significant differences among various clinicopathological factors of BRCA. There are also clear relationships between estrogen receptor, progesterone receptor, and human epidermal growth factor receptor-2, which are closely linked to the occurrence and development of BRCA (**Figure 8**).

### Tumor microenvironment analysis

**Figure 9:**
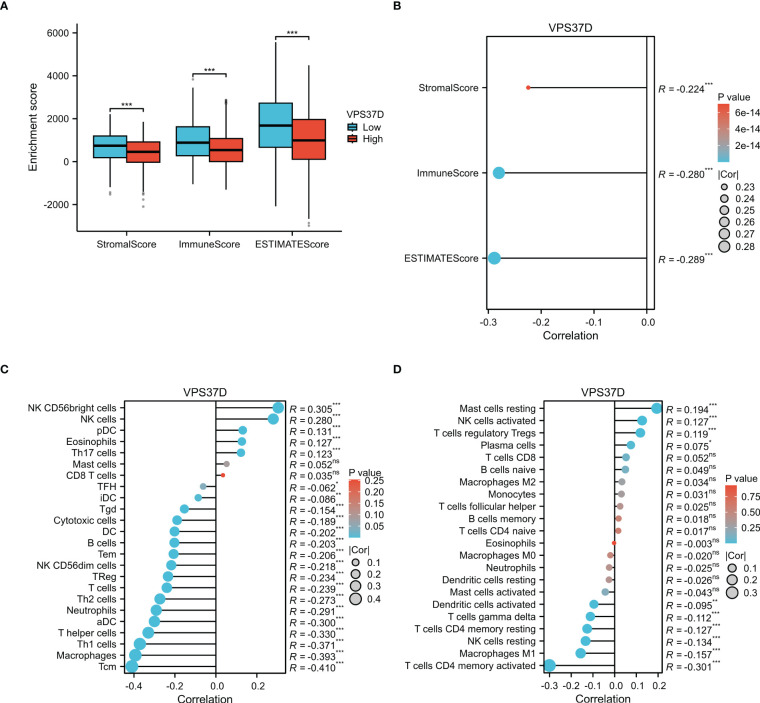
Analysis of the immune cell infiltration. **(A)** Differences in VPS37D expression levels in the immune score; **(B)** Immune infiltration score, matrix score, and estimated score were correlated with VPS37D expression; **(C)** The relationship between VPS37D expression obtained by the ssGSEA algorithm and immune cells; and **(D)** The association between VPS37D expression, as determined by the Cibersort algorithm, and immune cell populations (*P < 0.05, **P < 0.01, ***P < 0.001). ns, no significance.

To confirm whether VPS37D is involved in the immune regulation of BRCA, we performed immune cell infiltration analysis using a variety of immune scoring methods. The estimate analysis results showed that the immune cell score in the high VPS37D expression group was lower than that in the low expression group (*P <*0.05, **Figure 9A**). It was negatively correlated with immune score (*P <*0.05, **Figure 9B**). Additionally, ssGSEA analysis revealed that VPS37D expression was negatively correlated with immune cells, such as macrophages and central memory T cells (Tem) (*P <*0.05, **Figure 9C**). Subsequently, we performed cross-validation using the CIBERSORT analysis, and the results were generally consistent. CIBERSORT analysis revealed significant differences in VPS37D expression among resting mast cells, activated NK cells, regulatory T cells (Tregs), CD4 memory activated T cells, CD4 resting memory T cells, and resting NK cells, with an inverse relationship with macrophages M1 (*P <*0.05, **Figure 9D**). These results suggest that VPS37D may regulate the occurrence and development of BRCA by influencing immune cell infiltration and immune responses.

## Discussion

The ESCRT machinery is a crucial regulator of membrane remodeling and cellular signaling, and plays a complex role in oncogenesis (47). This study systematically revealed the molecular characterization of ESCRT family genes across 33 cancer types using integrated multi-omics data from TCGA database for the first time. Significant differences in the expression of ESCRT members were observed in BLCA, LIHC, and THYM. VPS37D was upregulated in BRCA and GBM, whereas it was significantly downregulated in KIRC, indicating a tissue-specific regulatory role. Prognostic association specific to cancer: Survival analysis indicated that elevated CHMP4B expression was correlated with extended survival in OV patients, whereas it was significantly linked to unfavorable prognosis in gastric cancer STAD. The bidirectional regulation mode may arise from variations in the molecular pathways mediated by ESCRT across distinct tumor microenvironments. The expression level of ESCRT exhibited a significant negative correlation with CD8 ^+^ T cell infiltration and a positive correlation with PD-L1 expression across 16 cancer types. This finding supports the previously established mechanism by which ESCRT facilitates the secretion of exosomal immunosuppressive molecules in liver cancer studies.

ESCRT exhibits diverse regulatory characteristics across various cancers; however, its role is particularly significant in breast cancer. In the context of breast cancer, a deep prediction analysis revealed that differential expression of the VPS37D gene has a significant impact on disease prognosis and progression. The present study showed that VPS37D exhibits elevated expression levels in tumors, and increased expression is associated with worse patient prognosis. This gene is significantly associated with multiple clinical factors and surface receptors that are typical of breast cancer. Immune infiltration analysis indicated that immune cell scores in the VPS37D high-expression group were reduced, with expression levels closely correlating with the transformation of M1 macrophages. VPS37D is an important component of the ESCRT-I complex in the ESCRT pathway, and is expressed in various tissues and cell types, with functions related to the transport and degradation of intracellular proteins. Although studies on the relationship between VPS37D and cancer are relatively limited, existing studies have shown that VPS37D frameshift mutations (incidence 7.1%) were detected in microsatellite unstable (MSI)-type gastric cancer and colorectal cancer, indicating that mutations disrupt the membrane-binding capacity of the ESCRT-I complex and affect endosomal protein sorting, confirming for the first time that VPS37D is a potential driver gene for digestive tract tumors (48). At the same time, VPS37D exhibits chromosomal structural variation. RNA sequencing of single cells showed that VPS37D expression decreased by 65% during neuronal differentiation. This suggests that VPS37D may help control synaptic vesicle transport (49). In the keratoconus model, VPS37D and STAM 1 levels dropped by 40% (50). CRISPR knockdown of VPS37D led to an abnormal increase in the diameter of multivesicular bodies, and overexpression of VPS37D partially restored the corneal stromal cells’ ability to respond to mechanical stress. Together, these three studies demonstrated the importance of VPS37D in the membrane transport system. It is a key part of the ESCRT-I complex (amino acid residues 121–187 are responsible for ALIX binding), and problems with its function can cause a wide range of diseases, from tumor growth to tissue breakdown.

This suggests that VPS37D plays an important role in tumorigenesis and progression. Modulation of the expression of VPS37D or other genes within the ESCRT family may result in abnormal ESCRT functions, which could affect tumor cell survival and proliferation. These findings indicate that ESCRT may be a viable target for tumor treatment. Interference with the functions of ESCRT complexes in cells may inhibit tumor cell growth and dissemination. Moreover, utilizing the spatial and functional properties of ESCRT within cells could enhance the creation of innovative targeted therapeutic agents or biomarkers for improved tumor diagnosis and treatment.

Nonetheless, the study’s limitations include insufficient data on certain rare cancers, such as uveal melanoma; *in vitro* validation requires supplementation; and the interaction mechanism between ESCRT and cellular metabolism remains unclear. Future efforts will concentrate on: ① the development of small-molecule inhibitors aimed at the ESCRT complex assembly interface; ② the validation of the ESCRT scoring system’s application value in immunotherapy stratification utilizing a multicenter cohort. In summary, ESCRT functions as a critical mechanism for intracellular protein sorting and transport, contributing to various aspects of tumor biology. ESCRT is expected to offer significant opportunities for future research and clinical applications in tumor treatment.

## Conclusions

ESCRT family genes vary in tumors and influence immune cell infiltration, prognosis, and chemotherapy efficacy. In breast cancer, VPS37D correlates with clinicopathological factors and the tumor microenvironment. ESCRT may serve as a novel prognostic marker and immune target, although its regulatory mechanisms remain unconfirmed, potentially impacting tumor progression and prognosis. The regulatory mechanisms of the ESCRT family in tumors should be explored in the future through experimental validation.

## Data Availability

Publicly available datasets were analyzed in this study. This data can be found here: https://www.cancer.gov/ccg/research/genome-sequencing/tcga.

## Supplementary material

Supplementary Figure 1 The division of labor among the various subunits of ESCRT.

Supplementary Figure 2 Differential expression of ESCRT family genes in the CPTAC database.

Supplementary Figure 3 Prognostic analysis of ESCRT in pan-cancer.

Supplementary Figure 4 Correlation of ESCRT expression with immune subtypes.
