# Supplementary figures and images for: ESCRT may function as a tumor biomarker, transitioning from pan-cancer analysis to validation within breast cancer
Source: Front Immunol. 2025 Mar 31;16:1531940. doi: 10.3389/fimmu.2025.1531940 (PMC11994712; doi:10.3389/fimmu.2025.1531940)

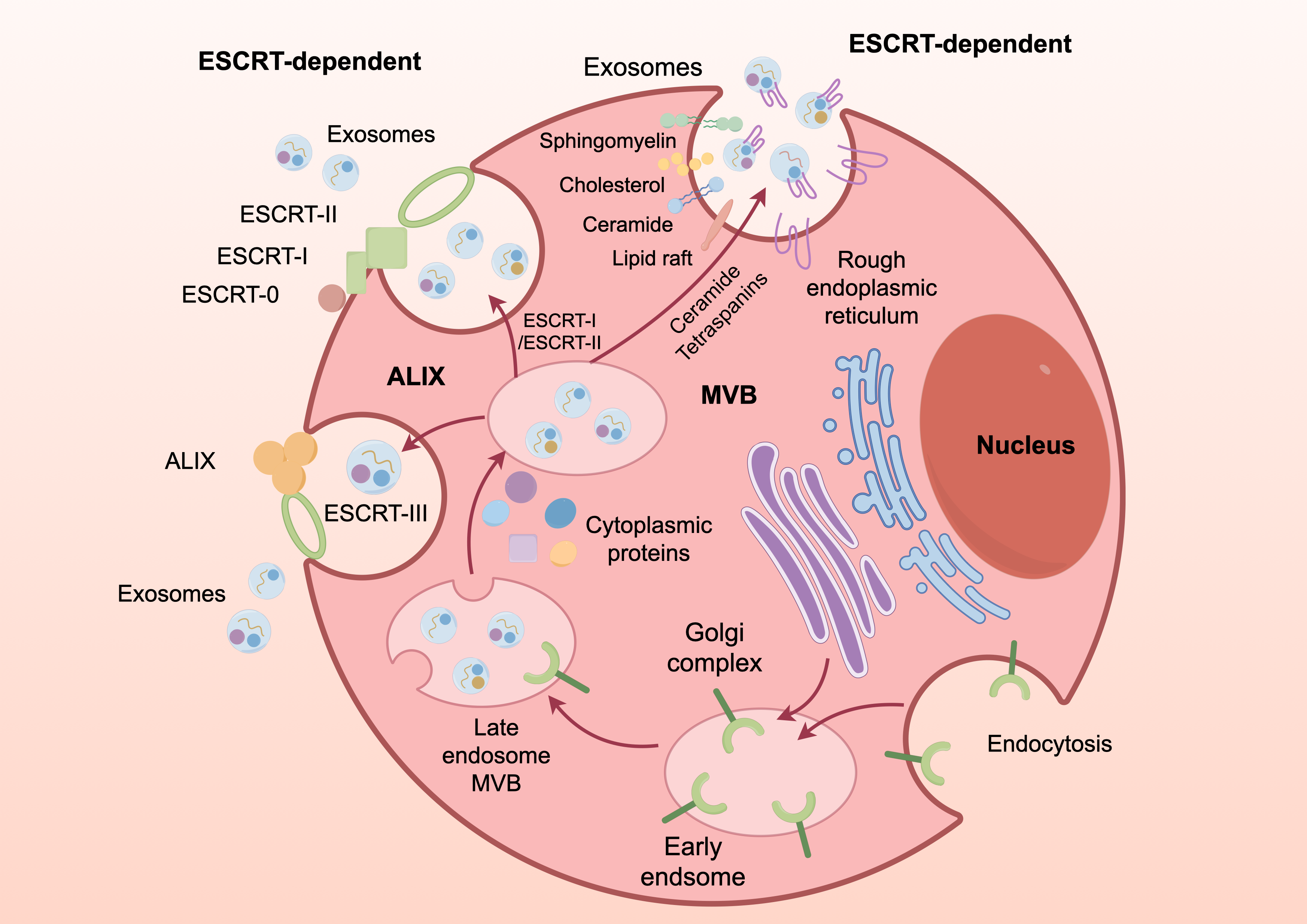

Supplement: Supplementary Figure 1 — The division of labor among the various subunits of ESCRT. [file Image1.tiff]

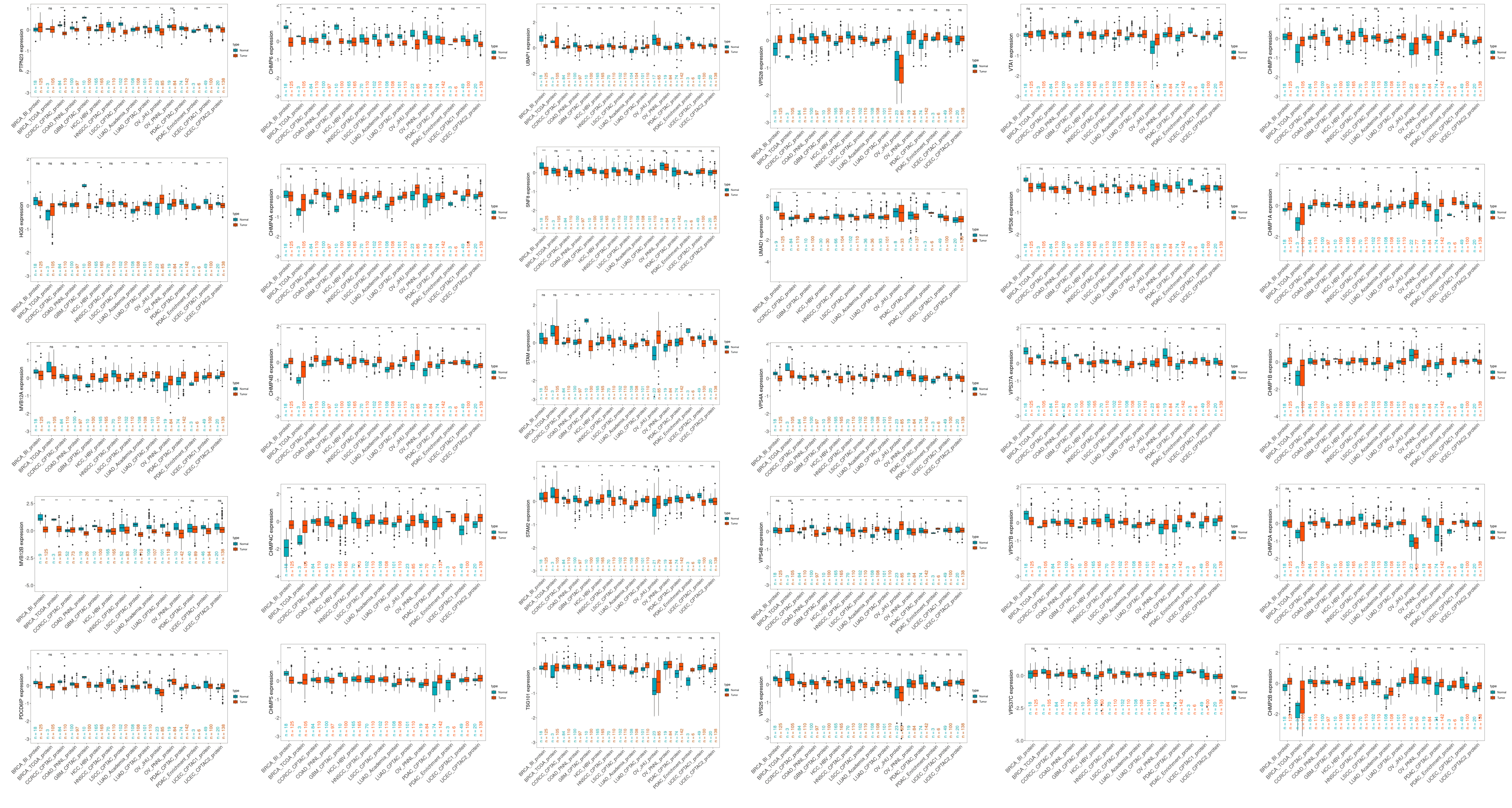

Supplement: Supplementary Figure 2 — Differential expression of ESCRT family genes in the CPTAC database. [file Image2.png]
