# Supplementary material for: ESCRT may function as a tumor biomarker, transitioning from pan-cancer analysis to validation within breast cancer
Source: Front Immunol. 2025 Mar 31;16:1531940. doi: 10.3389/fimmu.2025.1531940 (PMC11994712; doi:10.3389/fimmu.2025.1531940)
Supplement: Supplementary file 5 [file Presentation1.pdf]

Captions for Supplementary Figures

Supplementary Figure 1 | The division of labor among the various subunits of ESCRT.
Supplementary Figure 2 | Differential expression of ESCRT family genes in the CPTAC database.
Supplementary Figure 3 | Prognostic analysis of ESCRT in pan-cancer
Supplementary Figure 4 | Correlation of ESCRT expression with immune subtypes;
